# Retraction: An Indole Alkaloid from a Tribal Folklore Inhibits Immediate Early Event in HSV-2 Infected Cells with Therapeutic Efficacy in Vaginally Infected Mice

**DOI:** 10.1371/journal.pone.0318042

**Published:** 2025-01-17

**Authors:** The PLOS One Editors

After this article [1] was published, concerns were raised about Fig 2. Specifically, panels A, E, and H appear similar to panels A, E, and H in Fig 6 of [2] despite representing different experimental conditions.

The corresponding author apologized for the error in Fig 2 and provided a corrected figure. They did not provide the raw image files or individual level data underlying the published results, as the corresponding author has superannuated from service in 2021 and the lab is closed.

In addition, during editorial follow-up it came to PLOS’ attention that there was an undisclosed competing interest between the Academic Editor and the corresponding author. In light of this issue, an independent member of the *PLOS One* Editorial Board reassessed the article. During the reassessment of the article, the Editorial Board member raised concerns that the reported methodology is inadequate and that the article includes several statements that are either incorrect or insufficiently supported by the literature. In addition, the Editorial Board member raised concerns about the welfare of the animals used in this study following their review of the reported methodology, particularly those related to the animal welfare evaluations. The authors do not agree and commented that the study was conducted following CPCSEA guidelines.

In light of the above concerns, which call into question the overall reliability of the results and conclusions reported in the published article, the *PLOS One* Editors retract this article.

All authors did not agree with the retraction.
